# Prevalence and trends of the diabetes epidemic in South Asia: a systematic review and meta-analysis

**DOI:** 10.1186/1471-2458-12-380

**Published:** 2012-05-25

**Authors:** Ranil Jayawardena, Priyanga Ranasinghe, Nuala M Byrne, Mario J Soares, Prasad Katulanda, Andrew P Hills

**Affiliations:** 1 Institute of Health and Biomedical Innovation, Queensland University of Technology, Brisbane, QLD, Australia; 2 Diabetes Research Unit, Department of Clinical Medicine, Faculty of Medicine, University of Colombo, Colombo, Sri Lanka; 3 Department of Pharmacology, Faculty of Medicine, University of Colombo, Colombo, Sri Lanka; 4 Curtin Health Innovation Research Institute, School of Public Health, Curtin University, Perth, Australia; 5 Mater Mother’s Hospital, Mater Medical Research Institute and Griffith Health Institute, Griffith University, Brisbane, QLD, Australia

**Keywords:** Diabetes Mellitus, South Asia, Epidemiology, Prevalence, Trends, Risk factors

## Abstract

**Background:**

Diabetes mellitus has reached epidemic proportions worldwide. South Asians are known to have an increased predisposition for diabetes which has become an important health concern in the region. We discuss the prevalence of pre-diabetes and diabetes in South Asia and explore the differential risk factors reported.

**Methods:**

Prevalence data were obtained by searching the Medline® database with; ‘prediabetes’ and ‘diabetes mellitus’ (MeSH major topic) and ‘Epidemology/EP’ (MeSH subheading). Search limits were articles in English, between 01/01/1980–31/12/2011, on human adults (≥19 years). The conjunction of the above results was narrowed down with country names.

**Results:**

The most recent reported prevalence of pre-diabetes:diabetes in regional countries were; Bangladesh–4.7%:8.5% (2004–2005;Rural), India–4.6%:12.5% (2007;Rural); Maldives–3.0%:3.7% (2004;National), Nepal–19.5%:9.5% (2007;Urban), Pakistan–3.0%:7.2% (2002;Rural), Sri Lanka–11.5%:10.3% (2005–2006;National). Urban populations demonstrated a higher prevalence of diabetes. An increasing trend in prevalence of diabetes was observed in urban/rural India and rural Sri Lanka. The diabetes epidemicity index decreased with the increasing prevalence of diabetes in respective countries. A high epidemicity index was seen in Sri Lanka (2005/2006–52.8%), while for other countries, the epidemicity index was comparatively low (rural India 2007–26.9%; urban India 2002/2005–31.3%, and urban Bangladesh–33.1%). Family history, urban residency, age, higher BMI, sedentary lifestyle, hypertension and waist-hip ratio were associated with an increased risks of diabetes.

**Conclusion:**

A significant epidemic of diabetes is present in the South Asian region with a rapid increase in prevalence over the last two decades. Hence there is a need for urgent preventive and curative strategies .

## Background

Diabetes mellitus has reached epidemic proportions worldwide, placing a substantial burden on healthcare services. Historically, diabetes was considered a disease confined to developed countries and affluent people. However, recent estimates suggest that the prevalence of diabetes is rising globally, particularly in developing countries [1]. South Asia, commonly known as the Indian sub-continent, is home to almost one-quarter of the world’s population and is comprised of many diverse ethnic, linguistic and religious groups. India, Pakistan, Bangladesh, Nepal, Sri Lanka, Bhutan and Maldives are the countries of the region. South Asians are known to have an increased predisposition for Type 2 diabetes [2]. In addition to the large population living in South Asia, a significant number of immigrants from the region are living in affluent Western nations. For example, the 2001 UK census reported that around 4.0% (2.3 million) of the country’s total population were of South Asian origin [3]. As a consequence, a disease such as Type 2 diabetes affecting the ethnic South Asian sub-population will have potential implications on global health.

Diabetes mellitus has become an important health concern in the South Asian region with an estimated increase in the prevalence of diabetes of over 151% between year 2000 and 2030. In the same period, diabetes is projected to increase by 40% [1]. Studies have consistently demonstrated that South Asians are at an increased risk of developing diabetes in comparison to other ethnic groups [2]. In the UK, the risk of diabetes is five times higher for immigrants from Pakistan and Bangladesh and three times higher for Indian immigrants, with an associated increased risk of complications, morbidity and mortality compared with the native white Caucasian population [4]. Furthermore, South Asian patients with diabetes were younger and less obese compared to the native white Caucasians [4]. Progression of diabetes is also known to be more rapid among South Asians and Mukhopadhyay et al. [5] reported that the decline in glycaemic control over time was much more rapid among South Asians when compared to Europeans. Hence, it is apparent that diabetes among South Asians represents a significant health concern with differential risk factors and a more aggressive progression than in other ethnic groups.

Although there have been comprehensive reviews on diabetes in the Asian region [6], among South Asians immigrants living in developed countries [7] and from individual South Asian countries such as India [8], to date no studies have explored the prevalence and trends of the diabetes epidemic for the South Asian region. The present study aims to discuss the prevalence of pre-diabetes and diabetes among adults from individual countries in the South Asian region and explore the differential factors reported to be associated with the development of diabetes in these countries.

## Methods

The study was conducted in adherence to the PRISMA (Preferred Reporting Items for Systematic Reviews and Meta-Analyses) guidelines and the PRISMA checklist is provided as a Supplementary file ( Additional file 1). Diabetes prevalence data among South Asian adults in regional countries were obtained by a three-stage process. In the first stage a search of the online Medline® database (Medical Literature Analysis and Retrieval System) was performed with a combination of MeSH® (Medical Subject Headings) terms; ‘prediabetes’ and ‘diabetes mellitus’ were the MeSH major topic and Epidemiology/EP was the MeSH subheading. The search limits were; language (‘English’), dates (between ‘1^st^ January 1980’ and ‘31^st^ December 2011’), Species (‘Humans’) and age (‘all adults: 19+ years’). The conjunction of the above results were narrowed down by adding the name of each regional country (India, Pakistan, Bangladesh, Sri Lanka, Nepal, Bhutan and Maldives), South Asian and Indian Asians as key words. In the second stage the total hits obtained from searching Medline® using the above search criteria were screened by reading the ‘title’ and ‘abstract’. Studies not satisfying the inclusion criteria were excluded at this stage. The studies selected for inclusion in stage two were further screened for suitability during stage three by reading the selected manuscripts. At this stage studies were excluded based on the following exclusion criteria: being confined to only a specific age group, being hospital/clinic-based, studies reporting the results of larger studies as duplications and studies conducted among South Asians residing elsewhere. To obtain additional data a manual search was performed using the reference lists of selected articles. This process was conducted by two independent reviewers (RJ and PR) and the final group of articles to be included in the review was determined after an iterative consensus process among the reviewers.

For the purpose of describing prevalence data for the individual countries, the studies that were most recent, nationally representative or with the largest sample size were included. For meaningful comparisons of prevalence data among the countries, age-standardized data are presented here, unless otherwise stated. Additional data not available in the published manuscript such as gender and area-specific prevalence were obtained from corresponding authors or calculated using the available raw data. Area of residence and social status are key factors determining the prevalence of diabetes; therefore, when exploring the secular trend in diabetes prevalence it is meaningless to plot the prevalence data from studies based on different populations even in a single country. Hence, when evaluating the secular trends in prevalence of diabetes and pre-diabetes in a country we only considered studies that evaluated the temporal change in prevalence between similar populations or prospective studies based on the same population.

Presence of ‘diabetes mellitus’ in the individual studies were defined according to the World Health Organization (WHO) or American Diabetes Association (ADA) criteria adopted at the time of the respective studies. ‘Prediabetes’ was defined as the presence of Impaired Fasting Glucose (IFG) or Impaired Glucose Tolerance (IGT) according to the above criteria. The diabetes epidemicity index (a prognostic index of the diabetes epidemic in a population) was defined as the ratio between the prevalence of IGT/IFG (pre-diabetes) and Total Glucose Intolerance (TGI) (diabetes and pre-diabetes) i.e. the ‘diabetes epidemicity index’ is the percentage of the TGI made up by IGT/IFG [9].

A meta-analysis of studies examining the risk factors associated with diabetes mellitus in South Asian populations was conducted for parameters that were defined identically across studies. Hence the meta-analysis was performed on the following clinical and biochemical parameters; family history of diabetes, age, male gender, Body Mass Index (BMI), Waist-Hip Ratio (WHR), Systolic Blood Pressure (SBP) and Diastolic Blood Pressure (DBP). A fixed effect analysis was initially conducted for all comparisons. Heterogeneity was assessed using the *χ*2 test on Cochrane’s Q statistic and by calculating I^2^. If significant heterogeneity was present (p < 0.05 from *χ*2 test) a random effects meta-analysis was carried out. Data were analysed using RevMan version 5.1.2 (Review Manager, Copenhagen: The Nordic Cochrane Centre, The Cochrane Collaboration, 2011) statistical software package. In all analyses a p-value < 0.05 was considered statistically significant.

## Results

**Figure 1:**
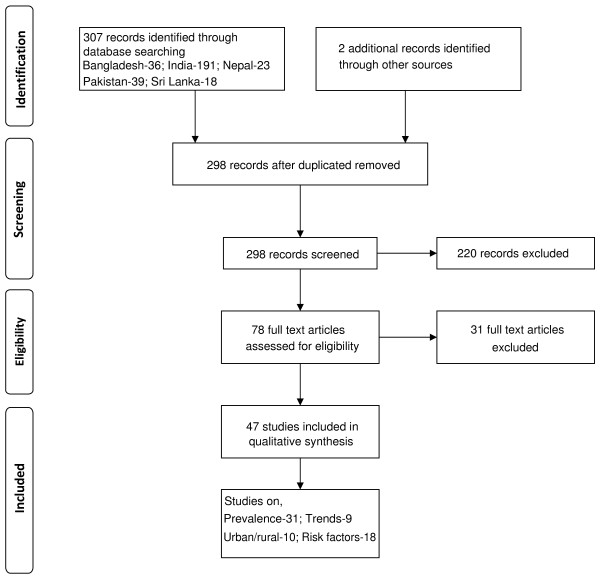
Summarized search protocol.

The number of articles identified using the above methodology for individual South Asian countries are summarized in Figure 1. However, we were unable to identify any published data for Bhutan. The International Diabetes Federation’s estimated prevalence for diabetes in Bhutan for 2010 was 3.6% [10].

### Prevalence of diabetes and pre-diabetes

The prevalence of diabetes and pre-diabetes in the respective countries and the sample characteristics are summarized in Table 1. We were able to identify studies evaluating the prevalence of diabetes and pre-diabetes for each South Asian country however nationally representative surveys were only available for India [11], Pakistan [12] and Sri Lanka [13]. Most surveys reported the prevalence of pre-diabetes for all adults and both males and females, however there is a considerable heterogeneity in the prevalence, depending on the country, area of residence and study date. The Maldives STEP survey reported the lowest pre-diabetes prevalence of 3.0% (M: 2.3%, F: 3.7%) despite being a relatively recent study conducted in an urban population [14], and it was similar to results observed in rural Pakistan in 2002 [15]. The reported prevalence of pre-diabetes elsewhere in South Asia also showed a wide variation from 4.1% in urban India [16] to 19.5% in urban Nepal [17].

**Table 1:** Prevalence of diabetes and pre-diabetes in South Asian countries

	**Study date**	**Study setting [reference]**	**Sample size**	**Age group**	**Prevalence of pre-diabetes**			**Prevalence of Diabetes**			**Diabetes Epidemicity Index**	**Diagnostic criteria**
					**All**	**Male**	**Female**	**All**	**Male**	**Female**		
Bangladesh	1999**–**2000	Rural [18]	4923	≥20	12.4	12.7	12.1	3.8	5.2	3.4	76.5%	ADA
	2002	Rura [19]	1119	≥20	8.4	7.3	9.4	6.4	7.4	5.5	56.8%	ADA
	2004	Semi-urban [20]	3981	≥20	5.8	4.4	6.7	6.8	7.3	6.5	46.0%	WHO 1997
	2002**–**2003	Urban [21]	5265	≥20	5.2	4.7	5.5	10.5	10.4	9.9	33.1%	WHO 1997
	2004**–**2005	Rural[22]	975	≥20	4.7^†^	3.9	5.2	8.5^†^	9.4	8.0	35.6%	WHO 1997
	2000	Urban [23]	11216	≥20	14.0	14.6	14.3	12.1	12.5	11.9	53.6%	WHO 1997
	2000	Urban [24]	10025	≥20	8.1	8.4	7.9	13.9	13.3	14.3	36.8%	WHO 1997
India	1999**–**2002	National [11]	18363	≥25	5.2	5.6	5.5	4.3	4.4	4.5	54.7%	WHO 1997
	2002**–**2003	Urban [25]	10930	20**–**69	5.3	6.2	3.9	10.1	11.1	8.4	34.4%	WHO 1997
	2002**–**2005	Urban [16]	986	>18	4.1	4.3	4.1	9.0	8.7	9.2	31.3%	WHO 1997
	2005	Rural [26]	4535	≥30	15.5	16.6	14.3	13.2	14.3	12.0	54.0%	ADA
	2007	Rural [27]	1645	≥20	4.6	5.4	4.9	12.5	16.5	13.5	26.9%	WHO 1997
India	2008**–**2009	Urban [28]	2227	≥20	13.2	NR	NR	11.1	NR	NR	54.3%	WHO 1997
	2009**–**2010	Rural [29]	1370	≥20	12.0	10.5	13.6	19.8	16.1	22.0	37.7%	WHO 1997
Maldives	2011^a^	Rural [30]	1266	≥20	NR	NR	NR	10.3	8.4	12.0	NR	ADA
	2004	National [14]	1556	25**–**64	3.0	2.3	3.7	4.5	4.3	4.7	40.0%	WHO 1997
	1999**–**2001	Urban and rural [31]	1841	≥20	6.5^†^	7.0^†^	6.1^†^	10.6^†^	11.6^†^	9.8^†^	38.0%	ADA
Nepal	2001**–**2002	Urban [32]	1012	≥40	11.5	13.2	10.2	8.5	10.8	6.9	57.5%	ADA, WHO 1997
	2007^a^	Urban [17]	740	≥20	19.5	25.0	15.0	9.5	11.8	7.9	67.2%	ADA
Pakistan	1995	National [12]	5433	≥25	10.2^†^	6.6^†^	12.1^†^	8.7^†^	9.3^†^	11.1^†^	54.0%	WHO 1994
	2002	Rural [15]	2032	≥25	3.0	4.2	2.3	7.2	10.1	4.3	29.4%	ADA
	2000	Urban [33]	1042	30**–**64	NR	NR	NR	6.5	5.0	6.6	NR	ADA
Sri Lanka	2000**–**2001	Urban and rural [34]	6047	30**–**65	14.1^†^	14.2	14.1	13.8^†^	14.2	13.5	50.5%	ADA, WHO 1997
	2005**–**2006	National [13]	4532	≥20	11.5	11.0	11.7	10.3	9.8	10.9	52.8%	ADA, WHO 1997

† calculated from available data; NR – Not reported; a - publication year.

The prevalence of diabetes also demonstrated a wide variation between countries. In Bangladesh no studies were based on a nationally representative sample, however, regional surveys in urban and semi-urban populations showed a moderately high prevalence of diabetes (6.8%–10.5%) [20,21]. In rural Bangladeshi populations the prevalence of diabetes which was 3.8% in 1999–2000 [18], had increased to 8.5% by 2004–2005 [22]. In India, many studies have explored the prevalence of diabetes with estimates varying considerably between different geographical areas and between urban and rural populations. The Prevalence Of Diabetes in India Study (PODIS) reported an age-standardized prevalence of 4.3%, 4.4% and 4.5% for all adults, and males and females, respectively [11]. However, more recent studies based on urban populations or rapidly developing regions have reported a higher prevalence of diabetes (10.1%) [16,25] while other studies from rural Indian populations have demonstrated an even higher prevalence (12.5%–13.2%) [26,27].

Results from the STEPS survey conducted in urban Male, Maldives showed a 4.5%, 4.3% and 4.7% prevalence of diabetes in all adults, males and females, respectively [14]. A survey conducted in urban Nepal between 2001 and 2002 showed that 10.8% and 13.2% of males suffered from diabetes and pre-diabetes respectively, with the values for females being 6.9% and 10.2%, respectively [32]. According to the Pakistan National Diabetes Survey (PNDS), 9.3% males and 11.1% females suffered from diabetes in 1995 [12] and a rural survey showed a higher proportion of males were affected by diabetes (10.1%) but not females (4.3%) [15]. No recent data were available regarding the present situation and to explore the current trends in Pakistan. A nationally representative diabetes and pre-diabetes study in Sri Lanka showed that the age-standardized prevalence of diabetes in Sri Lankan adults was 10.3% [males 9.8%, females 10.9%, P > 0.05][13], while a population-based survey conducted in four of the nine Sri Lankan provinces reported a prevalence of 14.2% and 13.5% of diabetes among males and females, respectively [34]. In 2000, a regional survey in a Sri Lankan suburb (Maharagama) showed that 6.5% of all adults, 5.0% of males and 6.6% of females were affected by diabetes [33]. Hence, according to studies published in the last two decades in South Asia, the prevalence of diabetes showed a wide variation between 3.8% [18] in rural Bangladesh to 13.9% urban India [24].

### Prevalence of diabetes in south Asian urban and rural populations

The Maldives STEP survey was conducted in the country’s main commercial center, Male with no prevalence data available for the rural sector [14]. National or regional studies from other South Asian countries demonstrate a substantial difference in diabetes prevalence between urban and rural populations with the prevalence consistently being higher among urban residents (Table 2). We evaluated the degree of difference between the respective urban and rural prevalence data by calculating an Urban:Rural prevalence ratio. The ratio for Bangladesh was 3.5 [35], while for India it varied from 1.2–2.4 [11,36-39]. Pakistan demonstrates one of the lowest ratios of 1.4 [12], followed by Sri Lanka 1.9 [13]. Nepal demonstrated the highest ratio for urban and rural difference in the prevalence of 5.8 [31]. In short, there is strong evidence suggesting a higher prevalence of diabetes among urban South Asians.

**Table 2:** Prevalence of diabetes according to area of residence

**Country [reference]**	**Year**	**Urban sector**			**Rural sector**			**Urban:Rural ratio**
		**All**	**Males**	**Females**	**All**	**Males**	**Females**	
Bangladesh[35]	2005^a^	8.1	7.7	8.5	2.3	1.9	2.5	3.5
India[36]	1998^a^	5.9	7.0	5.0	2.9	3.0	2.7	2.0
India[37]	1996**–**1998	2.2	2.6	1.7	1.8	1.8	1.8	1.2
India[11]	1999**–**2002	5.6	5.6	5.8	2.7	2.5	2.5	2.1
India[38]	2003**–**2005	7.3	NR	NR	3.1	NR	NR	2.4
India[39]	2005**–**2007	13.5	14.0	10.2	6.2	5.6	5.9	2.2
Nepal[31]	1999**–**2001	14.6	14.9	14.3	2.5	4.1	1.2	5.8
Nepal[40]	2005**–**2006	22.8	NR	NR	20.0	NR	NR	1.1
Pakistan[12]^†^	1995	10.5	11.6	10.3	7.6	8.3	7.4	1.4
Sri Lanka[13]	2005**–**2006	16.4	NR	NR	8.7	NR	NR	1.9

### Trends

**Figure 2:**
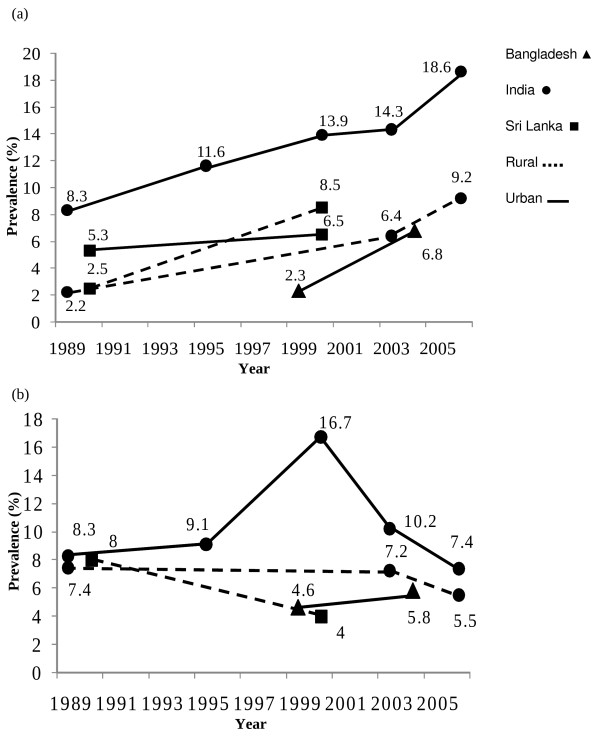
**Trends in prevalence in South Asia of a) diabetes mellitus and b) pre-diabetes (Data for individual countries were extracted from the following references; Bangladesh**[20]**; India**[41-45]**; Sri Lanka**[33,46,47]**).**

Studies evaluating secular trends in the prevalence of diabetes and prediabetes were available only for Bangladesh [20], India [41-45] and Sri Lanka [33,34,46,47]. The prevalence of diabetes in an urban Indian population has significantly increased from 8.3% in 1989 to 18.6% in 2005, and during the same period a similar increase from 2.2% to 9.2% was observed in a rural Indian population (p < 0.001)[45]. Similarly, a study in Sri Lanka demonstrated that the age-standardized prevalence of diabetes had significantly increased from 2.5% in 1990 to 8.5% in 2000 (p < 0.01) in a rural community [46], with only a slight increase in urban Sri Lanka from 5.3% to 6.5% during the same period [33,47]. These findings are summarized in Figure 2a. The trends for the prevalence of pre-diabetes are not as definitive, for example, the increased prevalence observed in urban India in the period 1989 (8.3%) to 2000 (16.7%) had declined to 7.4% by 2006. Prevalence data in rural populations of India and Sri Lanka also showed a decline in prevalence during a similar period (Figure 2b). An increase in the prevalence of pre-diabetes has been observed among urban Bangladeshi populations during the last decade.

### The diabetes epidemicity index

**Figure 3:**
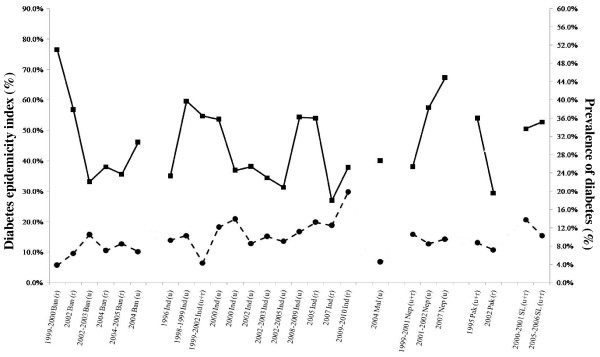
**Diabetes epidemicity index of South Asian countries.** (Ban – Bangladesh; Ind – India; Mal – Maldives; Nep – Nepal; Pak – Pakistan; SL – Sri Lanka; u – urban; r – rural; u + r – urban and rural; Diabetes [**·**]; Diabetes Epidemicity Index [▪]).

The temporal change in the diabetes epidemicity index and prevalence of diabetes in the regional countries are shown in Figure 3. The epidemicity index decreases as the prevalence of diabetes increases in the respective countries [9]. The most recent available data suggests a high epidemicity index in Sri Lanka (2005/2006 – 52.8%) and urban India (54.3%), while for other countries in the region for which recent data are available, the epidemicity index is comparatively low (rural India 2007 – 37.7% and urban Bangladesh – 33.1%).

### Risk factors

**Figure 4:**
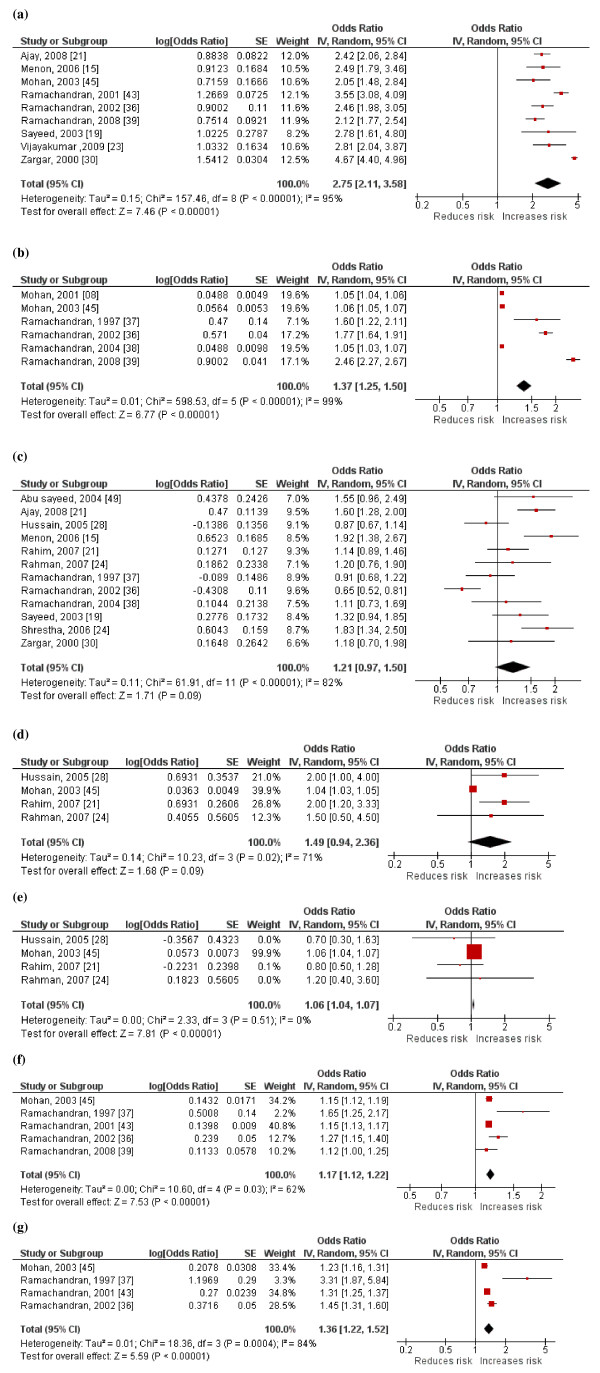
Forest plot showing pooled odds ratios for a) Family history, b) Age, c) Male gender, d) Systolic Blood Pressure, e) Diastolic Blood Pressure, f) Body Mass Index and g) Waist-Hip ratio associated with diabetes (IV-Inverse variance; SE-Standard Error).

A forest plot of the studies evaluating risk factors associated with diabetes among South Asians is shown in Figure 4. The pooled odds ratio from random effects analysis for family history is 2.75 (95% CI: 2.11, 3.58; p < 0.001). The significant overall effect indicates that family history is a significantly associated risk factor for diabetes (Figure 4a). The forest plot for age also shows a similar distinct increase in risk of diabetes with increasing age (Figure 4b). Male gender does not demonstrate a similar distinct pattern, the increased risk shown by several studies have been contradicted by others (Figure 4c). The forest plot of SBP demonstrates a significant increase in risk of diabetes with increasing SBP (Figure 4d). In contrast DBP does not show a similar distinct pattern (Figure 4e). Increasing BMI (Figure 4f) and WHR (Figure 4g) were both associated with a significantly increased risk of diabetes.

## Discussion

This is the first comprehensive report to systematically evaluate the scientific literature on the prevalence, trends and risk factors for diabetes in the South Asian region. Prevalence, based on the most recent national surveys in the countries of the region were; 4.5% in Maldives (2004) [14], 4.3% in India (1999–2002) [11], 8.7% (1995) in Pakistan (1995) [12] and 10.3% in Sri Lanka (2005–2006) [13]. However, it is noteworthy that more recently published data from India indicates a much higher prevalence of 9.2% (rural) and 18.6% (urban) in 2006 [45]. In addition, recent reports also highlight a secular increase in prevalence in the region. Hence, it is apparent that despite the differences in methodology, diagnostic criteria and age of subjects studied, the region is facing an epidemic of diabetes. This is more evident when the observed prevalence is compared with available data from other regions (Table 3), with observed prevalence comparable to recent global and regional estimates by the International Diabetes Federation (2011) [10].

**Table 3:** Prevalence of diabetes in different regions

**Region**	**Year**	**Prevalence of Diabetes**
South Asia*	1995**–**2005/2006	4.5%**–**10.3%
Global†	2011	8.5%
Middle-East†	2011	11.0%
North America†	2011	10.7%
South America†	2011	9.2%
South-East Asia†	2011	9.2%
Western Pacific†	2011	8.3%
Europe†	2011	6.7%
Africa†	2011	4.5%

* based on most recent national surveys in regional countries

† IDF 2011 [10].

The increased prevalence of diabetes in the South Asian region could be attributed to regional changes in disease patterns from communicable to non-communicable diseases [48]. The reasons attributed to this shift in disease pattern include: increased life expectancy, rapid population growth, unplanned urbanization, low literacy and increased external debt with resultant cutbacks on national healthcare expenditure [48]. Collectively, these and related issues have contributed to the emergence of non-communicable diseases such as diabetes as a substantial regional health problem. This so-called ‘epidemiological transition’ could also be linked to the rapid industrialization occurring in the region as evidenced by the high prevalence of diabetes among urban residents [49]. It is important to note that this epidemiologic transition and the rate of increase in non-communicable diseases such as diabetes in developing countries is far greater than that previously observed in high income countries, and hence there is a need to find solutions in a much shorter time frame and with far fewer resources [50]. Recent national level data from Maldives indicates a very low prevalence of both diabetes and pre-diabetes despite approximately two-thirds of the population being overweight, the highest in the region [14]. Maldives is an island nation in the Indian Ocean and relatively isolated from the rest of the region, and with an economy based on tourism and the fishing industry. Hence it is debatable whether diabetes in the Maldives presents as a different disease entity compared to the rest of the South Asian region or differential exposure to risk factors/healthy lifestyles have resulted in a low prevalence. This difference merits further investigation.

The high prevalence of pre-diabetes observed in many South Asian countries highlights a potential indicator of further progression of the epidemic in the region. The combined prevalence of diabetes and prediabetes (IGT/IFG), i.e. total glucose intolerance (TGI), may serve as a useful measure of the public health impact of the epidemic. It has also been postulated that the ‘diabetes epidemicity index’ (% of the TGI made up by IGT/IFG) has a predictive value in determining the stage of an epidemic of glucose intolerance in a given population [9]. Our results also bear evidence to this fact as demonstrated by the decrease in the ‘epidemicity index’ in the different countries with progressive secular increase in the frequency of diabetes increases (Figure 3). Hence with the prevalent diabetes epidemic in the region at present the recent ‘epidemicity indices’ for most regional countries are relatively low. However, it is noteworthy that the present prevalent epidemic in the region had been preceded by a high ‘epidemicity index’. Hence strategies aimed at primary prevention could be helpful to ameliorate a further increase in the diabetes epidemic in populations such as Sri Lanka where recent data shows a high prevailing ‘epidemicity index’.

Family history, age, male gender, BMI, WHR, systolic and diastolic blood pressure were significant risk factors for diabetes among South Asians. In addition, few studies have also demonstrated an association between diabetes and wealth/income [21,23,27,42], hypercholesterolemia [27], physical inactivity [21,23,38,43], the presence of acanthosis nigricans [16], graduate education [45] and office-based occupation [23]. A meta-analysis could not be performed for these risk factors due to the limited number of studies or due to variations in definitions/classifications of risk factors between studies. The recent epidemic of diabetes in the region could be primarily due to environmental factors such as diet and physical activity levels coupled with a genetic predisposition [7,51]. The strong evidence for the association between diabetes and family history highlights a genetic contribution to the prevalent epidemic [52]. In addition, in this ethnically diverse population, increasing age and body weight have also been demonstrated as important contributory factors. This is evident by the association between diabetes and increasing BMI, waist-hip ratio and abdominal obesity [16,22,23,27]. This may be the cause of the high susceptibility for diabetes and other metabolic abnormalities among South Asians [7].

People in the South Asia have faced under-nutrition for many generations; they are born smaller however coupled with subsequent obesity increases risk for insulin resistance syndrome in later life [53]. A recent review has reported several dietary factors associated with insulin resistance among South Asians, such as higher intakes of carbohydrate, saturated fatty acids, trans-fatty acids and n-6 PUFA, and lower intakes of n-3 PUFA and fiber, hence the Asian diet may be an important contributory factor for the high disease prevalence [51]. During recent years urbanization has risen unprecedentedly in the South Asian region [48]. There are unhealthy lifestyle changes that are known to be associated with urbanization such as the lack of physical activity, changes in dietary habits and stress, all of which increases the risk of diabetes, as evidenced by the association shown in many South Asian studies. Rural-to-urban migration was also found to be a major risk factor for diabetes and obesity among South Asians [39]. Migrants changed their lifestyles considerably within a decade and physical activity status quickly reached urban levels acquiring a metabolic risk similar to that of urban dwellers [39]. Furthermore, our results also highlight that the levels are rising in rural South Asian communities [44,46]. Increased mechanization of the agriculture industry, automation of daily activities, popularization of television and increased computer usage in rural areas are leading to changes in lifestyle with resultant decrease in physical activity.

An intra-urban disparity in the prevalence of diabetes has also been reported in India [54]. In contrast to developed countries, socially-deprived urban South Asians reported relatively lower prevalence of diabetes and general obesity compared to their affluent counterparts [4]. Ramachandran et al. reported that age-standardized prevalence of diabetes and impaired glucose tolerance were significantly lower in the low income urban dwellers compared to an affluent group in a similar residential area [42]. This observation could be partly explained by the differential purchasing ability with the affluent having a higher ability to purchase food, increasing energy intake and obesity; while on the other hand, the less affluent people are more likely to engage in manual labour increasing their physical activity level. However, socially deprived diabetes patients demonstrate a poor glycaemic control, which is likely to be lack of access to proper health care facilities and relative lack of knowledge.

There were several limitations identified in the studies that this review is based upon; all South Asian prevalence studies reported the prevalence of diabetes with no distinction made between the different types of diabetes. Therefore this data could represent the sum of both type 1 and 2 diabetes. However, unlike in Europe, South Asians have a considerably lower level of type-1 diabetes (1–2%) and thus these prevalence data closely resemble the total prevalence of type 2 diabetes [1]. In addition, all studies included in our review were community-based surveys. Hence, this data may be an underestimate of the true regional burden, since a significant proportion of patients with diabetes may well be admitted in hospitals and care centers. Moreover, some studies have reported the prevalence of only known diabetes. The definitions and diagnostic criteria have also changed over the last two decades influencing prevalence rates. However, for the purpose of describing prevalence data for the individual countries the studies that were most recent were included. Hence the variations in diagnostic criteria are likely to be minimal as older studies were excluded. In addition when evaluating secular trends (Figure 2) we have used studies that were on the same population and used the same diagnostic criteria. The definition of pre-diabetes also varies between studies, with some studies using only IFG [16,55], some IGT [37,42] and some using both [13]. There is also a heterogeneity in analytical methods as some studies applied capillary blood and glucometers whereas others used venous blood and sophisticate biochemical analysis.

## Conclusions

In conclusion, our review highlights a significant epidemic of diabetes in the South Asian region with a rapid increase in prevalence over the last two decades. It is evident that several modifiable and non-modifiable risk factors play an important role in the pathogenesis of diabetes among South Asians. Hence there is a need for urgent preventive and curative strategies to be implemented.

## Competing interest

The author(s) declare that they have no competing interests.

## Authors’ contributions

RJ, PR, NMB, MJS, PK and APH made substantial contribution to conception and study design. RJ and PR were involved in data collection. RJ, PR, NMB, MJS and APH were involved in refining the study design, statistical analysis and drafting the manuscript. NMB, MJS, APH and PK critically revised the manuscript. All authors read and approved the final manuscript.

## Pre-publication history

The pre-publication history for this paper can be accessed here:

http://www.biomedcentral.com/1471-2458/12/380/prepub

## Supplementary Material

Additional file 1 PRISMA 2009 checklist. Click here for file
